# Alfaxalone and Dexmedetomidine as an Alternative to Gas Anesthesia for Micro-CT Lung Imaging in a Bleomycin-Induced Pulmonary Fibrosis Murine Model

**DOI:** 10.3389/fvets.2020.588592

**Published:** 2020-10-08

**Authors:** Erica Ferrini, Laura Mecozzi, Luisa Corsi, Luisa Ragionieri, Gaetano Donofrio, Franco Fabio Stellari

**Affiliations:** ^1^Department of Veterinary Science, University of Parma, Parma, Italy; ^2^Department of Medicine and Surgery, University of Parma, Parma, Italy; ^3^Chiesi Farmaceutici S.p.A., Corporate Pre-Clinical R&D, Parma, Italy

**Keywords:** anesthesia, Alfaxalone, Dexmedetomidine, pulmonary fibrosis, micro-CT imaging, mice, lung, bleomycin

## Abstract

Micro-CT imaging could be considered a powerful non-invasive tool for accessing pulmonary fibrosis in mice. However, the choice of the anesthesia protocol plays a fundamental role to obtain robust and reproducible data, avoiding misinterpretations of the results. Inhaled anesthesia is commonly used for micro-CT lung imaging, but sometimes the standardization of the protocol may be challenging for routine activities in drug discovery. In this study we used micro-CT to evaluate the effects of two anesthetic protocols, consisting in Alfaxalone and Dexmedetomidine mixture, as injectable agents, and gaseous isoflurane, on vehicle and bleomycin-treated mice. No significant differences were highlighted between the protocols either for lung aeration degrees by micro-CT or histologic analyses in both the controls and bleomycin-treated groups. Our results support Alfaxalone and Dexmedetomidine mixture as a suitable and safe alternative compared to isoflurane for lung imaging. We also concluded that this injectable mixture may be applied for several imaging technologies and on different mice models.

## Introduction

Computed Tomography (CT) imaging plays a key role for the diagnosis of several respiratory disorders in clinical practice (1). This technology provides a non-invasive evaluation of both the morphometry and functionality of lungs, exploiting the inherent contrast between air and tissue (2).

Recently, micro-CT imaging has been applied in some animal models of lung diseases including fibrosis and emphysema (3–5), emerging as a powerful tool for the longitudinal assessment of disease progression (2, 3, 6). However, translating CT technique to pre-clinical research has some challenging issues, namely due to the small size of rodents and their rapid respiratory cycle (3, 7). Moreover, an anesthesia protocol that ideally minimizes the interferences with the respiratory system studied is always necessary, to prevent undesirable animal movements avoiding the misinterpretations of functional results (8).

Inhaled anesthetics are commonly used in preclinical imaging due to their ability to rapidly induce sedation, to provide a real-time control on the depth of anesthesia and quick recovery time. Among its widespread applications in animal research, isoflurane is frequently chosen for longitudinal pulmonary imaging (9). Nonetheless, moderate respiratory and cardiovascular system depression and hypothermia (10, 11) can occur and expensive extra-equipment for gas delivery and scavenging systems to remove the excess gas are always required, thus hindering their diffusion. Further, technical limitations emerged using gas anesthesia during routine drug screening experiments aimed to investigate the efficacy of new potential anti-fibrotic compounds in a murine pulmonary fibrosis model. In fact, a constant monitoring is generally required to modulate the concentration of isoflurane and, thus, the depth of the anesthesia, making the process very time consuming, especially when a high number of mice is recruited.

A common alternative solution is represented by injectable ketamine-based combinations with α_2_-adrenergic agonists, such as xylazine. This anesthetic protocol is often used for surgical procedures in small animals, as well as for *in vivo* imaging. Anyway, the relatively prolonged recovery time and the relevant side effects like weight loss, hypothermia and respiratory depression (12) make this method unsuitable either for lung functional imaging or longitudinal studies, where repeated surveys are required in the same subject. Furthermore, ketamine is classified as narcotic and psychotropic drug, thus its use is often subjected to restrictions and require specific authorizations in accordance with national regulations.

Alfaxalone, a neuroactive GABA_A_-agonist steroid can be considered a safe alternative to ketamine, since it is not a restricted agent and it is extensively employed in veterinary science for its safety profile. Alfaxalone induces anesthesia at relatively low doses when associated with analgesic drugs such as xylazine or dexmedetomidine, and it is often used to achieve a surgical plan of anesthesia in mice and rats (13–15). Dexmedetomidine is, indeed, a more potent α_2_-agonist compared to xylazine, which guarantees myorelaxation avoiding popcorn-like jumping induced by the only injection of Alfaxalone (14) and it may be likewise reversed by the antagonist administration (atipamezole hydrochloride) (16).

To the best of our knowledge, the “injectable anesthetic protocol” has never been used for micro-CT imaging procedures, hence its application in this field has never been reported.

The aim of this study was to set up an anesthetic protocol based on Alfaxalone and Dexmedetomidine mixture, providing a safe and easy-to-use procedure compared to inhaled isoflurane gas anesthesia, in order to anesthetize healthy and fibrotic mice during micro-CT lung imaging.

## Materials and Methods

### Mice

The study was conducted using female inbred C57BL/6JOlaHsd mice (*Envigo, San Pietro al Natisone, Udine, Italy*) aged 7- to 8-weeks. Prior to use, mice were acclimatized for at least 5 days to the local vivarium conditions (20–24°C room temperature; 40–70% relative humidity; 12-h light-dark cycle), having free access to standard rodent chow and softened tap water. All animal experiments described herein were authorized by the official competent authority and approved by the intramural animal-welfare committee for animal experimentation of Chiesi Farmaceutici under protocol number: 841/2019-PR and comply with the European Directive 2010/63 UE, Italian D.Lgs 26/2014 and the revised “Guide for the Care and Use of Laboratory Animals” (17).

Pulmonary fibrosis was induced through the oropharyngeal aspiration (OA) (5) of 25 μg/mouse bleomycin hydrochloride (*Baxter, BLM*) diluted in 50 μl of saline, while only saline was instilled in vehicle mice. OA procedure was performed at day 0 and day 4 in mice previously anesthetized with 2.5% isoflurane.

### Pilot Study: Dose-Finding and Physiologic Monitoring

Ten Vehicle and 10 BLM mice were anesthetized with different doses of Alfaxalone (*Alfaxan*^®^*, Jurox Inc., Missouri, USA*) and Dexmedetomidine (*Dexdomitor*^®^*, Zoetis Inc., New Jersey, USA*), as reported in Table 1. The anesthetic solutions were prepared in saline and intraperitoneally (IP) injected 10 ml/kg of body weight. At day 7 the highest doses 60+0.5 mg/kg (*n* = 2 vehicle, *n* = 2 BLM) and 50+0.5 mg/kg (*n* = 2 vehicle, *n* = 2 BLM) were tested, followed by 40+0.5 mg/kg (*n* = 3 vehicle, *n* = 3 BLM) and 30+0.3 mg/kg (*n* = 3 vehicle, *n* = 3 BLM) at day 14. At day 21 all mice were re-anesthetized with the combination 30+0.3 mg/kg (*n* = 5 vehicle, *n* = 5 BLM) and 20+0.3 mg/kg (*n* = 5 vehicle, *n* = 5 BLM).

**Table 1:** Dose-finding study of Alfaxalone and Dexmedetomidine in C57BL/6JOlaHsd mice (*n* = 10 vehicle; *n* = 10 BLM).

**Alfa+Dex (mg/kg)**	Induction^*^ (min)	Duration^*^ (min)
60+0.5	1.5 ± 0.7	112.5 ± 10.6
50+0.5	2.3 ± 0.4	75.0 ± 7.1
40+0.5	2.6 ± 0.9	58.4 ± 5.9
30+0.3	3.0 ± 1.3	36.3 ± 6.0
20+0.3	n.a.	n.a.

* *Data are expressed as mean ± SD; n.a., not available*.

At each session, atipamezole hydrochloride (*Antisedan*^®^*, Zoetis Inc., New Jersey, USA*) 1.0 mg/kg was administered subcutaneously at least 20 min after the anesthesia induction, in order to rapidly reverse the α_2_-agonist activity. A wash out period of 7 days between two consequent administrations allowed mice to be completely recovered.

Some critical parameters were identified and monitored by micro-CT, such as the induction period (absence of the pedal withdrawal reflex), muscle relaxation, recovery time (time duration of anesthesia considering the administration of the atipamezole) and the maintenance of a stable breathing rate (100–120 brpm). In order to select the best dose of Alfaxalone (Alfa) and Dexmedetomidine (Dex) for micro-CT lung imaging, an induction time of 2–3 min and duration of anesthesia within 30 min were considered acceptable.

### Comparative Study

In this study, additional 54 mice were recruited for the comparison of the selected combination of Alfa+Dex (30+0.3 mg/kg) to gas anesthesia and instilled twice with vehicle (*n* = 20) or BLM (*n* = 34), according to the experimental design.

Three weeks after the first OA administration, which represents our common endpoint for lung fibrosis evaluation (5), mice were randomized into two distinct subgroups (Iso: *n* = 8 vehicle, *n* = 11 BLM; Alfa+Dex: *n* = 12 vehicle, *n* = 23 BLM).

All the mice underwent micro-CT imaging using one of the two anesthetic protocols: 2% isoflurane (*IsoFlo, Zoetis Inc., New Jersey, USA*) administered using PerkinElmer's XGI-8 Gas Anesthesia System and the IP injection of 30+0.3 mg/kg Alfa+Dex. After the imaging session mice were euthanized, BALF samples were collected and lungs were preserved for further histological analyses.

### Computed Tomography

**Figure 1:**
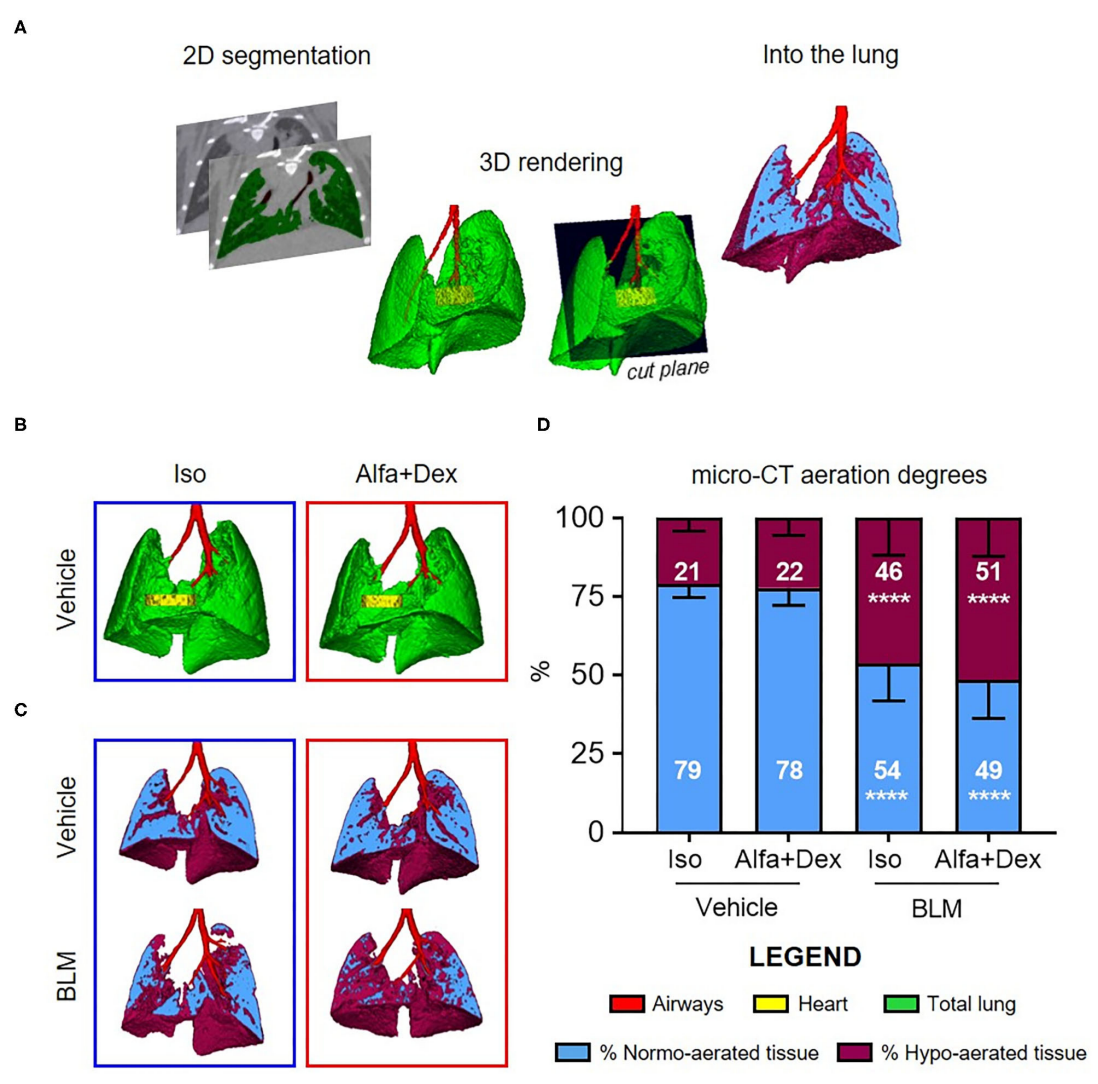
Micro-CT imaging to compare gas and injectable anesthesia protocols. **(A)** A schematic diagram of micro-CT images processing. **(B)** Representative 3D lung renderings of vehicle mice anesthetized alternatively with Isoflurane (*blue box*) or Alfa+Dex (*red box*) at day 21 after the first oral aspiration procedure. Airways, heart and total lung are colored in *red, yellow*, and *green*, respectively. Some representative cutting planes for vehicle and BLM mice showing the distribution of normo- and hypo-aerated compartments within each slice **(C)** and their quantification on total lung volumes **(D)**. Results are expressed as mean ± SD and changes in BLM groups were compared to vehicle using 2-way ANOVA followed by Sidak's test for multiple comparison; ****p < 0.0001.

Micro-CT imaging was performed with Quantum GX Micro CT (*PerkinElmer, Inc. Waltham, MA*). Images were acquired with an intrinsic retrospective two phase respiratory gating technique with the following parameters: 90 KV, 88 μA over a total angle of 360° for a total scan time of 4 min. The “high speed” scan mode resulted in two 3D datasets, corresponding to the two different phases of the breathing cycle (inspiration and expiration), but data reported here refer to the end-expiration phase. The reconstructed datasets were analyzed using Analyze software (*Analyze 12.0; Copyright 1986-2017, Biomedical Imaging Resource, Mayo Clinic, Rochester, MN*). A semi-automatic segmentation was used to define airways and total lung volumes. Micro-CT images were rescaled into Hounsfield units (HU), setting −1,000 HU as the density of air and 0 HU as the density of water. Pre-clinical HU density ranges (8) were applied to semi-automatically segmented lungs for the quantitative assessment of parenchymal lesions. Normo-aerated tissue [−860 HU; −435 HU] and hypo-aerated tissue [−435 HU; −121 HU] were defined and normalized on total lung volumes. The post-processing scheme is briefly described in Figure 1A. Breathing rate (brpm) was constantly recorded during image acquisitions.

### *Ex vivo* Analyses

Mice were euthanized by anesthetic overdose followed by bleeding from the abdominal aorta. Bronchoalveolar lavage fluid (BALF) was collected by gently washing the lungs with 0.6 mL sterile solution [Hank's balanced salt solution x10; ethylenediaminetetraacetic acid 100 mM; 4-(2-hydroxy-ethyl)-1-piperazineethansulphonic acid 1 mM; distilled water] for three times in the bronchial tree. The cell pellet was resuspended in 0.2 ml of PBS and white blood cells (WBC) were counted with an automated cell counter (*Dasit XT 1800J, Sysmex*).

Lungs were then excised and inflated with a cannula through the trachea by gentle infusion with 0.6 mL of 10% neutral-buffered formalin. After 24 h lungs were dehydrated in graded ethanol series, clarified in xylene and paraffin embedded. For each lung, three sections 5 μm thick were cut at 200 μm intervals, using a rotary microtome (*Slee Cut 6062, Slee Medical, Mainz, Germany*). Hematoxylin and eosin (H&E) and Masson's trichrome staining were performed for each slide. For analyses, slide images were acquired by NanoZoomer S-60 Digital slide scanner (*NanoZoomer S60, Hamamatsu, Japan*). Morphological changes in lung sections were graded semi-quantitatively according to the scale defined by Ashcroft (18) and modified by Hübner et al. (19) by two independent researchers blinded to the experimental design.

### Statistical Analysis

Statistical analyses were performed using Prism 8 software (*GraphPad Software Inc., San Diego, CA, United States*). The number of mice per group was defined by the GPower analysis using the G^*^Power version 3.1.9.4 software. All data were presented as mean ± SD. Two-way analysis of variance (ANOVA) was performed, followed by Sidak's multiple comparison *post-hoc* test to compare different experimental groups. Spearman correlation analysis was used to evaluate the relationship between histological parameters (i.e., Ashcroft score) and micro-CT outcomes (i.e., % hypo-aerated tissue). A *p* < 0.05 was considered as significant.

## Results

### Pilot Study

Vehicle and BLM mice were anesthetized with five different doses of Alfa and Dex, as reported in Table 1. Based on literature (20), we started from Alfa+Dex 60+0.5 mg/kg. This dose, the highest tested, was found to be unsuitable for lung imaging due to some observed side effects, such as the prolonged recovery time (112.5 ± 10.6 min) and the collapse of the left lobe in one vehicle mouse (Supplementary Figure 1). Since this alteration was not revealed anesthetizing the same animal with isoflurane, we speculated that Alfa+Dex 60+0.5 mg/kg could impair its lung function. The two intermediate doses of Alfa+Dex (50+0.5; 40+0.5 mg/kg) were not taken into account due to the long recovery times observed (75.0 ± 7.1; 58.4 ± 5.9 min, respectively). The lowest dose (20+0.3 mg/kg) was deemed inappropriate for imaging since it induced highly variable effects among treated mice, failing or provoking a very slight sedation and movement artifacts in micro-CT images, thus precluding the post-processing analyses. The combination of 30+0.3 mg/kg of Alfa+Dex was selected as the best dose for micro-CT lung imaging, showing a stable breathing rate (127 ± 7 brpm) within the 6–10 min after the imaging acquisition and a fast recovery time (36.3 ± 6.0 min). However, neither mortality nor differences were observed in vehicle and BLM groups, whether the doses tested.

### Comparative Study

The optimal dose of Alfa+Dex (30+0.3 mg/kg) was firstly compared to isoflurane (Iso, 2%) on saline-treated mice. No macroscopic differences between lungs were revealed from 3D renderings, as shown in Figure 1B.

Some representative images of vehicle and BLM lungs and the corresponding assessment of fibrosis are reported in Figures 1C,D, respectively. The lung aeration degrees of vehicle mice anesthetized with the two different protocols were strictly comparable (*p* > 0.99), with a prevalence (about 80%) of normo-aerated tissue in both cases. As expected, the double administration of BLM significantly affected the pulmonary gas exchange in BLM-treated mice compared to vehicle, increasing the % of hypo-aerated regions either for animals scanned using isoflurane (21 ± 4% vehicle vs. 46 ± 12% BLM; mean diff., 25; 95% CI, 11–40; *p* < 0.0001) or Alfa+Dex (22 ± 5% vehicle vs. 51 ± 12% BLM; mean diff., 29; 95% CI, 19–40; *p* < 0.0001) (Figure 1D). Neither in BLM nor vehicle mice were detected statistical differences in percentage of normo- and hypo-aerated tissues between both Alfa+Dex and Iso groups (*p* > 0.81).

**Figure 2:**
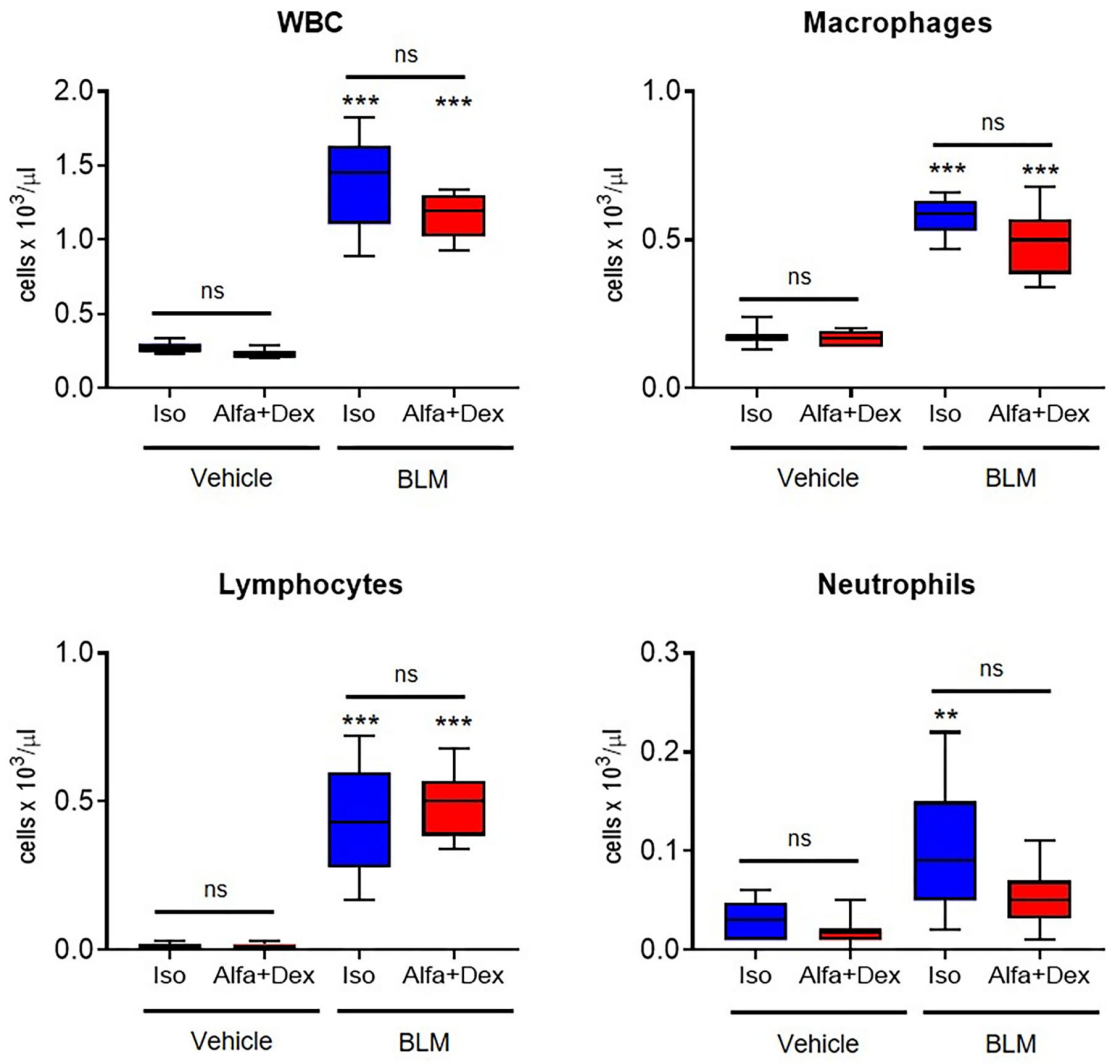
Inflammatory cells count in healthy and fibrotic BALF samples. Cellular infiltration in the BALF of vehicle and BLM groups (Iso, *blue*; Alfa+Dex, *red*) at 21 days after the first OA administration. Total white blood cells (WBC), macrophages, lymphocytes and neutrophils were expressed as cells per μL. Changes in BLM groups were compared to vehicle using 2-way ANOVA followed by Sidak's test for multiple comparison; **p < 0.01; ***p < 0.001.

Principal WBC populations have been measured, as reported in Figure 2. BALFs of vehicle mice contained a low amount of WBC and no significant differences have been revealed between the two anesthetic protocols, as well for the other cell populations. On the contrary, BLM groups showed a higher number of leucocytes compared to healthy mice, especially macrophages and lymphocytes which represent the prevalent inflammatory cells in the fibrotic parenchyma.

**Figure 3:**
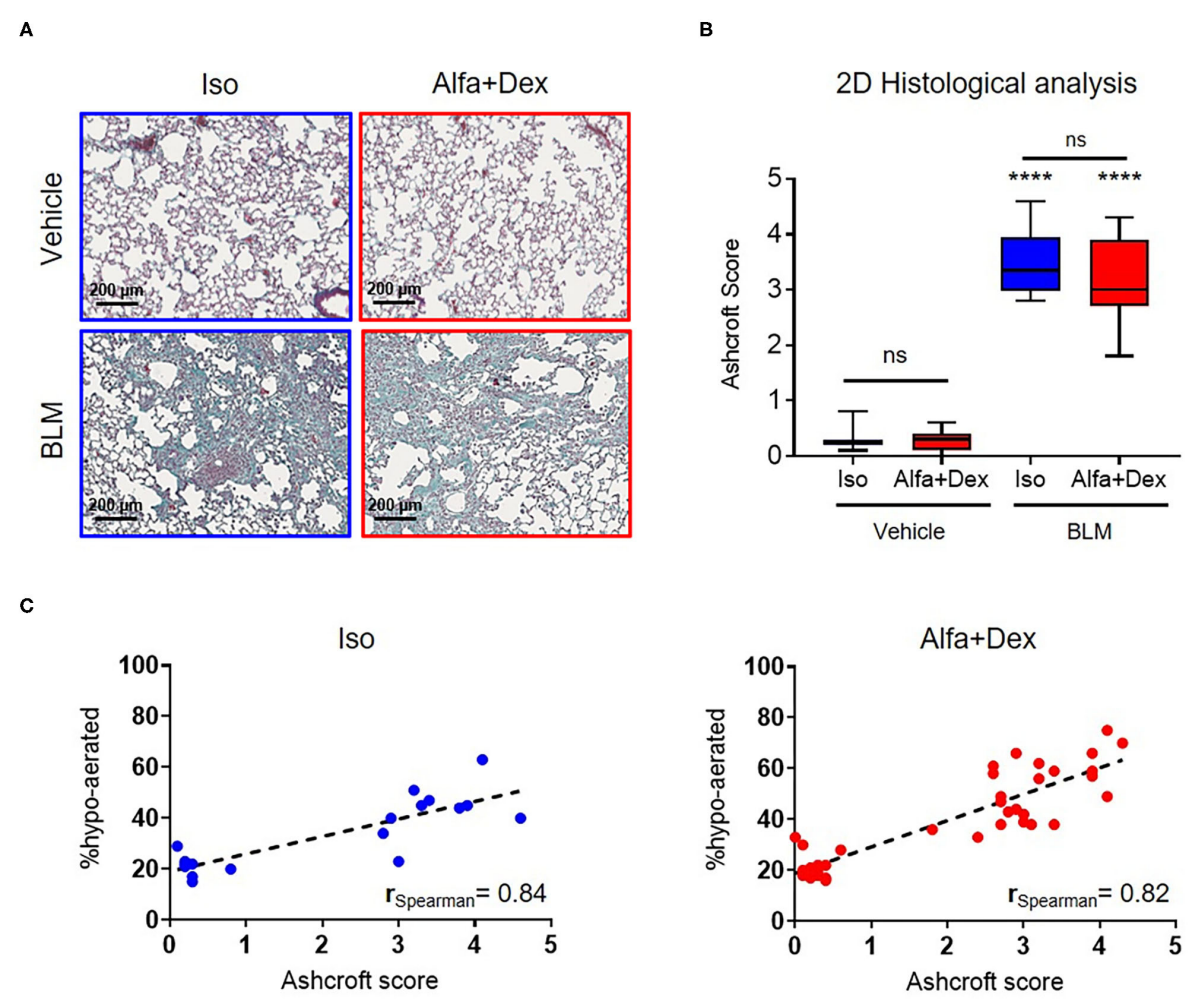
Gas vs. injectable anesthesia: histological outcomes and *in vivo* parameters. **(A)** Four representative images of Masson's trichrome stained sections of vehicle and BLM lungs (scale bar, 200 μm) 21 days after the first OA administration. **(B)** Ashcroft score variations in vehicle and BLM mice lungs comparing the two protocols of anesthesia. Changes in BLM groups were compared to vehicle using 2-way ANOVA followed by Sidak's test for multiple comparison; ****p < 0.0001. **(C)** Good Spearman correlations were found between histological Ashcroft score and % hypo-aerated tissue for Iso and Alfa+Dex protocols.

Some representative Masson's staining of vehicle and BLM lung slices are shown in Figure 3A. The mean Ashcroft score values for vehicle and fibrotic mice are reported in Figure 3B. A significant increase in Ashcroft score values in BLM groups compared to vehicle can be observed either for Iso (0.3 ± 0.2 Vehicle vs. 3.5 ± 0.6 BLM; mean diff., 3.2; 95% CI, 2.5–3.8; *p* < 0.0001) or Alfa+Dex (0.3 ± 0.2 Vehicle vs. 3.2 ± 0.6 BLM; mean diff., 2.9; 95% CI, 2.4–3.4; *p* < 0.0001) protocols. No differences were evidenced between the anesthetic groups for scale values. For all mice, linear correlations were performed comparing 3D micro-CT parameters (i.e., % hypo-aerated tissue) and 2D histological Ashcroft scores, resulting in r_Spearman_ = 0.84 (*n* = 19, *p* < 0.0001) and r_Spearman_ = 0.82 (*n* = 35, *p* < 0.0001) for Iso and Alfa+Dex anesthetized groups, respectively (Figure 3C).

## Discussion

High Resolution Computed Tomography (HRCT) is a powerful clinical tool for lung diseases diagnosis (1). The translation of this technology to pre-clinical studies can be crucial to better understand the pathology progression, minimizing the intra-experiment variation, thus reducing the number of mice used to reach the statistical significance.

Bridging this gap achieving robust and reproducible data is a challenging issue, since it requires well-characterized animal models and standardized micro-CT imaging protocols both for acquisition and post-processing steps. Unfortunately, clear guidelines are not available and the use of micro-CT in lung fibrosis drug discovery still limited.

To the best of our knowledge, different anesthetic protocols for lung imaging using micro-CT technology have never been compared.

Inhaled anesthetics actually represents the most common procedure for micro-CT lung imaging in mice, due to the quick induction and recovery time (9). However, physiological and technical limitations are associated to its use, such as moderate respiratory and cardiovascular system depression (10, 11).

We extensively used isoflurane anesthesia on different mouse strains and for several murine and rat models of pulmonary diseases (21). Anyway, as reported, several issues arose setting up a standardized gaseous anesthesia protocol for routine activities. In particular, BLM-treated mice were subjected to variable sedation times and, accordingly, to variable rates of gas exchange into the lungs, probably due to different stages of lung fibrosis lesions. This often caused unstable breathing rates and motion-related artifacts that hindering acquisitions and post-processing analyses of the images.

In our lab, gas anesthesia has also been tested on BALB/cOlaHsd mouse strain in a chronic asthma mouse model (22), experiencing the same critical issues described above. In addition, despite a successful acquisition, the analyses of micro-CT scans revealed a “hidden” collapse of the entire accessory right lobes in some sham BALB/cOlaHsd mice (Supplementary Figure 2).

These evidences forced us to explore other affordable injectable protocols of anesthesia, such as the combination of Alfa+Dex, since no dedicated equipment is required and the absence of exposure to noxious gases makes the procedure safe for personnel (23).

In this study, we found out the optimal concentration of Alfa+Dex mixture to anesthetize mice during micro-CT lung imaging, induce a stable breathing rate and shorten the induction (about 3 min) and recovery times (within 30 min).

The combination of Alfa+Dex was tested in comparison with isoflurane protocol in saline and BLM mice. The lung aeration degrees, lung parenchyma alterations and cellular infiltration in BALF were evaluated by micro-CT and *ex vivo* analyses, respectively. Micro-CT results revealed a significant increase in % of hypo-aerated tissue in BLM lungs compared to vehicle, likewise, for Isoflurane and Alfa+Dex, without any statistical difference between the two protocols of anesthesia. Histological Ashcroft score analyses corroborated micro-CT data, as lighted up by the high correlation coefficients obtained for both the anesthetics.

Moreover, the histological readouts and the count of cellular infiltration in BALF were evaluated in vehicle mice, used as controls to investigate the safety of Alfa+Dex mixture. No differences between gas and injectable protocols of anesthesia were revealed.

In the present study we successfully set up an injectable anesthesia protocol, as an alternative to isoflurane to perform micro-CT lung imaging.

Although we addressed our effort to investigate pulmonary fibrosis by micro-CT imaging, we believe that Alfa+Dex mixture may be employed for several imaging technologies (MRI, PET, BLI, etc.) on different mice models.

## Data Availability Statement

All datasets generated for this study are included in the article/Supplementary Material.

## Ethics Statement

The animal study was reviewed and approved by the intramural animal-welfare committee for animal experimentation of Chiesi Farmaceutici under protocol number: 841/2019-PR and comply with the European Directive 2010/63 UE, Italian D.Lgs 26/2014 and the revised Guide for the Care and Use of Laboratory Animals.

## Author Contributions

EF and FS: conception, design, and data collection. EF, LC, LR, and FS: laboratory testing. EF, LM, LC, LR, and FS: data analysis and interpretation. EF, LM, GD, and FS: drafting of manuscript. All authors contributed to the article and approved the submitted version.

## Conflict of Interest

LC and FS are employees of Chiesi Farmaceutici S.p.A., that supported the research work. The remaining authors declare that the research was conducted in the absence of any commercial or financial relationships that could be construed as a potential conflict of interest.

## Supplementary Material

Supplementary Figure 1 Alfa+Dex side effects by micro-CT. Representative coronal and transversal micro-CT lung sections and the corresponding 3D rendering for a vehicle C57BL/6JOlaHsd mouse anesthetized with the highest dose of Alfa+Dex tested (60+0.5 mg/kg). The absence of the whole left lobe is highlighted by three *blue arrowheads*.

Supplementary Figure 2 Micro-CT lung imaging on BALB/cOlaHsd mice anesthetized with isoflurane. 2.2% isoflurane was administered to induce and maintain anesthesia in *n* = 10 female BALB/cOlaHsd mice. Micro-CT imaging revealed the collapse of the accessory right lobe (*blue arrowheads*) in 5 of 10 mice.
