# Supplementary figures and images for: Alfaxalone and Dexmedetomidine as an Alternative to Gas Anesthesia for Micro-CT Lung Imaging in a Bleomycin-Induced Pulmonary Fibrosis Murine Model
Source: Front Vet Sci. 2020 Oct 8;7:588592. doi: 10.3389/fvets.2020.588592 (PMC7578219; doi:10.3389/fvets.2020.588592)

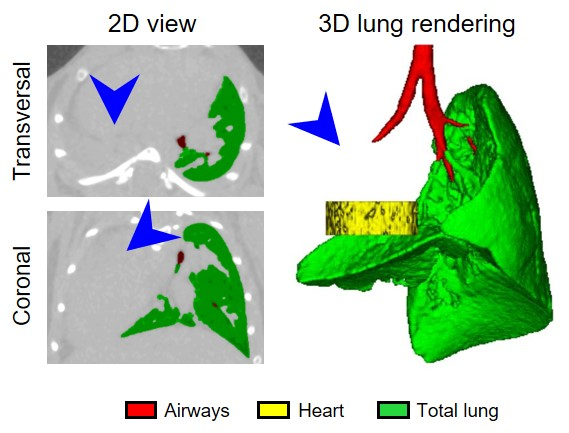

Supplement: Supplementary Figure 1 — Alfa+Dex side effects by micro-CT. Representative coronal and transversal micro-CT lung sections and the corresponding 3D rendering for a vehicle C57BL/6JOlaHsd mouse anesthetized with the highest dose of Alfa+Dex tested (60+0.5 mg/kg). The absence of the whole left lobe is highlighted by three blue arrowheads. [file Image_1.JPEG]

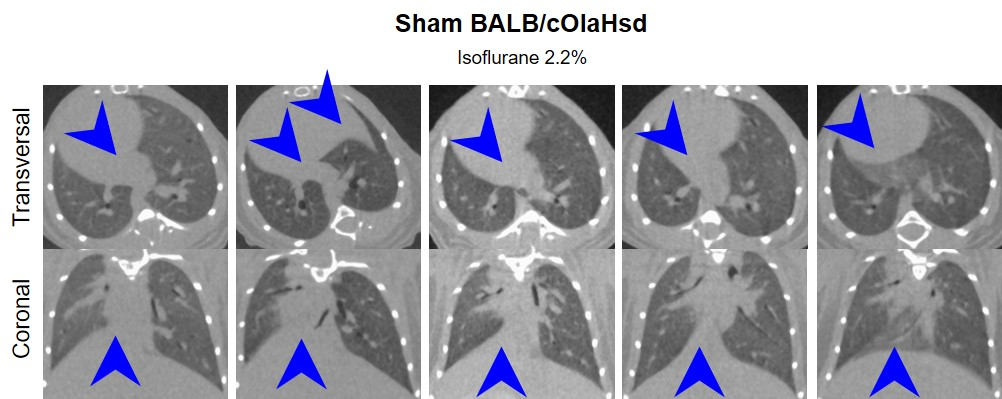

Supplement: Supplementary Figure 2 — Micro-CT lung imaging on BALB/cOlaHsd mice anesthetized with isoflurane. 2.2% isoflurane was administered to induce and maintain anesthesia in n = 10 female BALB/cOlaHsd mice. Micro-CT imaging revealed the collapse of the accessory right lobe (blue arrowheads) in 5 of 10 mice. [file Image_2.JPEG]
